# Application of Branched-Chain Amino Acids Mitigates Mitochondrial Damage to Spotted Seabass (*Lateolabrax maculatus*) Hepatocytes Cultured in High-Glucose and High-Fat Media

**DOI:** 10.3390/ani15040560

**Published:** 2025-02-14

**Authors:** Huijuan Ren, Yixiong Ke, Xueshan Li, Lin Wang, Kai Song, Francisco A. Guardiola, Chunxiao Zhang, Kangle Lu, Samad Rahimnejad

**Affiliations:** 1 State Key Laboratory of Mariculture Breeding, Fisheries College of Jimei University, Xiamen 361021, China; 18627101622@163.com (H.R.); xsli@jmu.edu.cn (X.L.); lingwang@jmu.edu.cn (L.W.); songkai@jmu.edu.cn (K.S.); zhcx29@163.com (C.Z.); 2 Immunobiology for Aquaculture Group, Department of Cell Biology and Histology, Faculty of Biology, Regional Campus of International Excellence “Campus Mare Nostrum”, University of Murcia, 30100 Murcia, Spain; faguardiola@um.es; 3 Key Laboratory of Biochemistry and Molecular Biology in Universities of Shandong (Weifang University), Weifang Key Laboratory of Coho Salmon Culturing Facility Engineering, Institute of Modern Facility Fisheries, College of Biology and Oceanography, Weifang University, Weifang 261061, China; 4 Weifang Key Laboratory of Salmon and Trout Health Culture, Conqueren Leading Fresh Science & Technology Inc., Ltd., Weifang 261205, China

**Keywords:** spotted seabass (*Lateolabrax maculatus*), hepatocytes, high-glucose or high-fat load, BCAAs, glucose and lipid metabolism, mitochondrial function

## Abstract

This study explored the metabolic effects of branched-chain amino acids (BCAAs) on the hepatocytes of spotted seabass (*Lateolabrax maculatus*) under high-glucose (HG) or high-fat (HF) conditions. Hepatocytes were cultured under five different conditions: control, high glucose (HG), HG + BCAAs (Leu 0.8 mM, Ile 0.4 mM, Val 0.8 mM), high fat (HF), and HF + BCAAs (Leu 0.8 mM, Ile 0.8 mM, Val 0.8 mM). After 72 h of culture, cells and cell supernatants were collected to measure relevant indicators. The results revealed that BCAAs supplementation significantly reduced glycogen and lipid accumulation in hepatocytes exposed to HG or HF conditions (*p* < 0.05). Additionally, alanine aminotransferase and aspartate aminotransferase activities in the supernatant were significantly decreased, indicating that BCAAs supplementation alleviated hepatocyte damage induced by these conditions. Furthermore, BCAAs addition markedly enhanced antioxidant defense by increasing superoxide dismutase and catalase activities, improving total antioxidant capacity, and reducing malondialdehyde levels. Metabolic enzyme activity analysis revealed that BCAAs significantly increased the activities of citrate synthase (CS), alpha-ketoglutarate dehydrogenase complex (α-KGDHC), succinate dehydrogenase (SDH), phosphoenolpyruvate carboxykinase (PEPCK), and liver pyruvate kinase (LPS), while significantly decreasing fatty acid synthase (FAS) activity. Gene expression analysis further demonstrated that BCAAs supplementation downregulated the expression of lipogenic genes (*fas* and *srebp-1c*) and upregulated the expression of lipolytic genes (*ppaα* and *atgl*) and glucose metabolism-related genes (*g6pd*, *hk*, *pfk*, *pk*, *fbp*, and *g6pase*). Under HG or HF conditions, hepatocytes exhibited decreased adenosine triphosphate (ATP) content, increased reactive oxygen species (ROS) levels, and reduced mitochondrial membrane potential. These adverse effects were mitigated by BCAAs supplementation. In conclusion, BCAAs supplementation alleviated hepatocyte damage caused by HG or HF conditions, enhanced antioxidant defenses, and protected mitochondrial activity and function by promoting glucose and lipid metabolism.

## Simple Summary

Mitochondria are central to cellular metabolism, energy production, and overall cell viability. In aquaculture, high-glucose or high-fat diets can induce oxidative stress, disrupt metabolic homeostasis, and impair mitochondrial function. Branched-chain amino acids (BCAAs), essential for animal growth, exhibit diverse biological benefits, including metabolic regulation and mitochondrial protection. This study demonstrated that BCAAs supplementation enhances mitochondrial function in hepatocytes of spotted seabass (*Lateolabrax maculatus*), effectively mitigating oxidative stress and cellular damage induced by excessive glucose or lipid exposure.

## 1. Introduction

Branched-chain amino acids (BCAAs), comprising leucine (Leu), isoleucine (Ile), and valine (Val), are essential amino acids that must be obtained through dietary sources [1,2]. BCAAs are among the most abundant amino acids in proteins and play pivotal roles in protein synthesis, as well as serving as carbon and nitrogen sources in metabolic pathways [3]. Maintaining a precise balance between BCAAs intake and metabolism is critical for physiological health.

Aberrant metabolism or incomplete oxidation of BCAAs, particularly Ile and Val, has been associated with catabolic stress and impaired mitochondrial function [4]. Multiple studies on mammals, including humans, have shown that feeding high-fructose diets combined with elevated BCAAs levels increased cardiac myocyte apoptosis in mice [5]. Similarly, excessive BCAAs intake has been linked to adverse effects such as insulin resistance and mitochondrial dysfunction [4,6]. Conversely, several other studies have highlighted the beneficial effects of BCAAs supplementation, including reduced muscle damage biomarkers and alleviated muscle soreness [7,8,9,10,11,12,13,14]. Diets with optimal BCAAs levels have also been shown to promote metabolic health in rodents and humans by enhancing hepatic insulin sensitivity, suppressing hepatic lipid accumulation, reducing protein catabolism, and increasing energy expenditure [15,16,17,18].

Spotted seabass (*Lateolabrax maculatus*) is a commercially important carnivorous marine fish in China. However, high-carbohydrate diets in carnivorous fish have been shown to induce liver glycogen accumulation, lipid deposition, hepatic damage, and disruptions in glucose metabolism [19,20,21]. Similarly, prolonged high-fat diets impaired lipid transport, compromised liver function, and increased the risk of fatty liver disease, posing a significant threat to fish health [22,23,24].

Given these metabolic challenges, spotted seabass serves as an ideal model for studying amino acid metabolism in fish. To better understand the metabolic effects of BCAAs in aquaculture species, this study investigated the impacts of BCAAs on mitochondrial function in spotted seabass hepatocytes under high-glucose or high-fat conditions. By using isolated hepatocytes, the study aimed to elucidate the metabolic response of farmed fish to BCAAs, providing valuable insights into the regulatory mechanisms of BCAAs metabolism in marine fish.

## 2. Materials and Methods

### 2.1. Model Establishment

The hepatocyte cell line used in this study was derived from spotted seabass hepatocyte, and established through tissue block migration and multiple passages during early culture stages and the cells were maintained in our laboratory under controlled conditions. Prior to experimentation, they were cultured in DMEM/F12 medium supplemented with 20% fetal bovine serum and 1% penicillin–streptomycin at 28 °C under 5% CO_2_ until reaching 70–80% confluence.

The experiment consisted of six treatment groups: control, high glucose (HG), HG + BCAAs (Leu 0.8 mM, Ile 0.4 mM, Val 0.8 mM), high fat (HF), and HF + BCAAs (Leu 0.8 mM, Ile 0.8 mM, Val 0.8 mM) groups, each with three replicates. The control group was maintained in standard DMEM/F12 medium, while the HG group was cultured in DMEM/F12 medium supplemented with 40 mM glucose [25]. The HF group was cultured in DMEM/F12 medium containing 0.1 mM fatty acids (prepared by mixing oleic acid and palmitic acid at a ratio of 1:1 from a 10 mM stock solution) [26] for 72 h. Following treatment, the supernatant was collected, and the cells were washed twice with PBS, harvested via trypsin digestion, and washed again with PBS before further analysis.

### 2.2. Measurement of Indicators

#### 2.2.1. Cellular Biochemical Analyses

Hepatocytes were seeded in 6-well cell culture plates and cultured until stable adhesion was achieved before initiating the experimental treatments. Following treatment, cells were collected for subsequent analyses. Glycogen content in each group was measured using Nanjing Jiancheng reagent kits (A043-1-1, Nanjing, China), following the manufacturer’s instructions.

Cell culture supernatants were collected and centrifuged at 4 °C at 1000 r/min for 10 min to separate the supernatant. Alanine aminotransferase (ALT) and aspartate aminotransferase (AST) levels were quantified using Nanjing Jiancheng reagent kits (Nanjing, China), following the provided protocols.

For lipid accumulation analyses, hepatocytes were seeded in 24-well cell culture plates and cultured until stable adhesion was achieved. After treatment, the culture medium was removed, and the cells were washed twice with PBS before fixation with Oil Red O fixative for 20–30 min. The fixative was then discarded, and cells were washed twice with distilled water. To prepare for staining, 60% isopropanol was added for 20–30 s and then discarded. Freshly prepared Oil Red O staining solution was applied for 10–20 min. After removing the staining solution, cells were washed with 60% isopropanol for 20–30 s until interstitial spaces were clear, followed by 2–5 washes with distilled water. For nuclear counterstaining, Mayer’s hematoxylin staining solution was applied for 1–2 min, then discarded, followed by 2–5 times washes with distilled water. Oil Red O buffer was applied for 1 min and then discarded. Finally, distilled water was added to cover the cells, and lipid accumulation was observed under an optical microscope (DM5000B, Leica, Wetzlar, Germany).

#### 2.2.2. Oxidative Stress-Related Indicators

##### Measurement of Antioxidant and Oxidative Stress Markers

Superoxide dismutase (SOD, A001-3-2) and catalase (CAT, A007-1-1) activities, malondialdehyde (MDA, A03-1-2) concentration, and total antioxidant capacity (T-AOC, A015-2-1) in hepatocytes were assessed using Nanjing Jiancheng (Nanjing, China) reagent kits according to the manufacturer’s instructions.

##### Measurement of Reactive Oxygen Species (ROS) Concentration

After cell collection, samples were washed twice with PBS. A DCFH-DA probe, diluted 1:1000 in serum-free culture medium, was added to the cells and incubated at 37 °C for 20 min. After incubation, the cells were washed three times with serum-free culture medium to remove excess probe. Finally, the ROS levels were quantitatively analyzed using a flow cytometer (CytoFLEX, Beckman Coulter, Suzhou, China).

#### 2.2.3. Key Enzymatic Activities in Hepatocyte Metabolism

The activities of citrate synthase (CS, BC1065), α-ketoglutarate dehydrogenase (α-KGDHC, BC0715), succinate dehydrogenase (SDH, BC0955), phosphoenolpyruvate carboxykinase (PEPCK, BC3315), and lipase (LPS, BC2345) in hepatocytes were measured using reagent kits from Beijing Solebao Technology Co., Ltd., (Beijing, China).

Fatty acid synthase (FAS, H231-1-1) activity and ATP (S0027) content in hepatocytes were assessed using reagent kits from Nanjing Jiancheng Co., Ltd., (Nanjing, China) and Biyun Tian Biotechnology Co., Ltd., (Shanghai, China), respectively.

#### 2.2.4. Mitochondrial Status

##### Mitochondrial Membrane Potential Detection

Cells were cultured in 6-well plates and allowed to adhere stably before undergoing experimental treatments. At the end of the experiment, the cells were collected and resuspended in 0.5 mL of cell culture medium. An equal volume (0.5 mL) of JC-1 staining working solution was added, and the mixture was gently inverted multiple times to ensure thorough mixing. The cells were then incubated at 37 °C in a cell culture incubator for 20 min. Following incubation, the cells were centrifuged at 600× *g* for 3–4 min at 4 °C, and the pellet was collected. The cells were washed twice with JC-1 staining buffer and resuspended in 1 mL of the same buffer. This process was repeated twice, involving centrifuging at 600× *g* for 3–4 min at 4 °C, discarding the supernatant, and resuspending the cells in JC-1 staining buffer. Finally, the cells were resuspended in an appropriate volume of JC-1 staining buffer and analyzed using a flow cytometer (CytoFLEX, Beckman Coulter, Pasadena, California, USA).

##### Mitochondrial Activity Staining

Cells were cultured on glass coverslips (cell climbing slices) and subjected to experimental treatments. Following treatment, the culture medium was removed, and the cells were incubated with Mito-Tracker Red CMXRos working solution (C1035, Beyotime, Shanghai, China) at 37 °C for 15–30 min. Following incubation, the Mito-Tracker Red CMXRos working solution was removed, and the cells were stained with culture medium containing 1X Hoechst 33,342 (C1027, Beyotime, Shanghai, China) live-cell staining solution. The cells were incubated at room temperature for 10 min. The staining solution was aspirated, and the cells were washed 2–3 times with PBS. An appropriate amount of anti-fluorescence quenching mounting medium was then applied, and a coverslip was placed over the cells. Finally, the samples were observed under a laser confocal microscope (TCSSP8, Leica, Wetzlar, Germany).

##### DNA Damage Detection

Cells were cultured on glass coverslip and subjected to experimental treatments. Following treatment, the medium was removed, and the cells were washed once with PBS. Then, 1 mL of fixing solution was added, and the cells were fixed for 5–15 min. After fixation, the fixing solution was aspirated, and the cells were washed three times with washing solution, each wash lasting 3–5 min. To block nonspecific binding, 1 mL of immunostaining blocking solution was added, and the cells were incubated at room temperature for 10–20 min. The blocking solution was then aspirated, and 1 mL of γ-H2AX mouse monoclonal antibody was applied. The cells were incubated at room temperature for 1 h. After incubation, the antibody solution was carefully removed, and the cells were washed three times with washing solution, each wash lasting 5–10 min. Next, 1 mL of anti-mouse 488 was added, and the cells were incubated at room temperature for 1 h. Following incubation, the secondary antibody was aspirated, and the cells were washed twice with washing solution, each wash lasting 5–10 min. For nuclear staining, 1 mL of DAPI staining solution was added, and the cells were stained at room temperature for approximately 5 min. The staining solution was then aspirated, and the cells were washed three times with washing solution, each wash lasting 3–5 min. Finally, an appropriate amount of anti-fluorescence quenching mounting medium was applied, and a coverslip was placed over the cells. The stained cells were then observed under a laser confocal microscope (TCSSP8, Leica, Wetzlar, Germany).

#### 2.2.5. Fluorescence Quantification

Total RNA was extracted using the Trizol method, following the manufacturer’s instructions. RNA concentration and purity were assessed using a microplate spectrophotometer (Thermo Scientific, Ayer Rajah, Singapore) at an absorbance ratio of 260/280 nm. RNA integrity was verified via 1% agarose gel electrophoresis. To eliminate genomic DNA contamination, RNA samples were treated with RQ1 RNase-Free DNase. cDNA synthesis was performed using the Novozyme kit (Tianjin, China), in accordance with the manufacturer’s protocol.

Real-time fluorescence quantitative PCR (qPCR) was conducted using the SYBR Green I fluorescence method on a QuantStudio Flex real-time PCR system (Thermo Scientific, Ayer Rajah, Singapore). The cycling conditions were set according to the kit instructions. Primer sequences were designed using Primer 5.0 software and synthesized by Shanghai Shenggong Biological Engineering Co., Ltd., (Shanghai, China). The relative expression levels of target genes were calculated using the 2^−ΔΔCt^ method, with β-actin as the reference gene. Primer amplification efficiency was validated for both the target and the reference genes. Each reaction was performed in triplicate to ensure accuracy. The primer sequences used in this study are listed in Table 1.

### 2.3. Data Analysis

Variance analysis was performed using SPSS version 23.0. Statistical significance was assessed through one-way ANOVA or *t*-test. Differences among the experimental groups were evaluated using Dunnett’s test for multiple comparisons. Results are presented as mean ± standard deviation (SD), with statistical significance set at *p* < 0.05.

## 3. Results

### 3.1. Cellular Biochemistry

**Figure 1:**
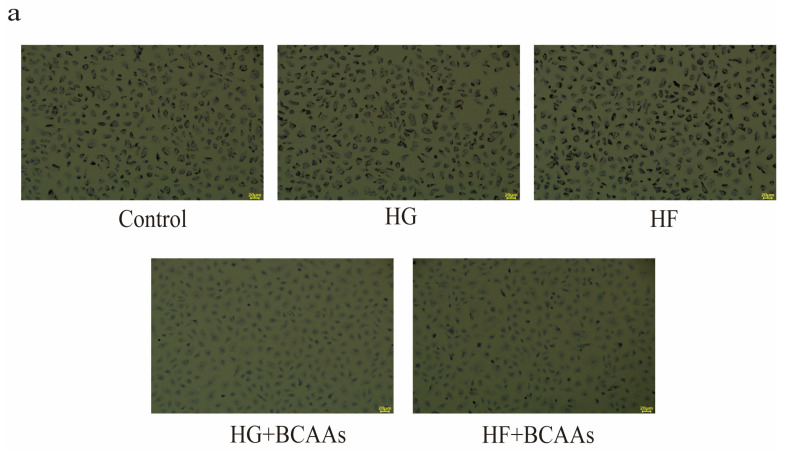

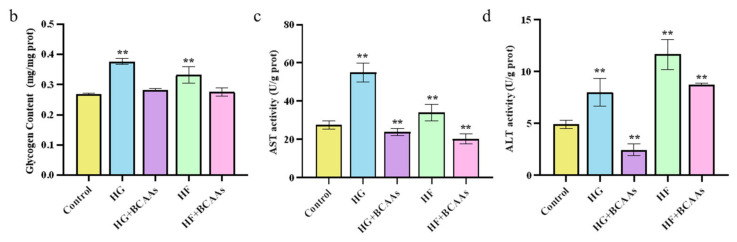
Effects of BCAAs supplementation on hepatocyte biochemistry in spotted seabass (*Lateolabrax maculatus*): (**a**) lipid deposition; (**b**) glycogen content; (**c**) ALT activity; (**d**) AST activity. ** indicates extremely significant differences between groups (*p* < 0.01).

BCAAs supplementation during cell culture effectively alleviated lipid droplet formation and reduced glycogen accumulation in hepatocytes induced by HG or HF exposure. Furthermore, ALT and AST activities in the culture medium were significantly lower in the BCAAs-treated groups compared to the HG and HF groups (*p* < 0.05; Figure 1).

### 3.2. Cellular Antioxidant Defense

**Figure 2:**
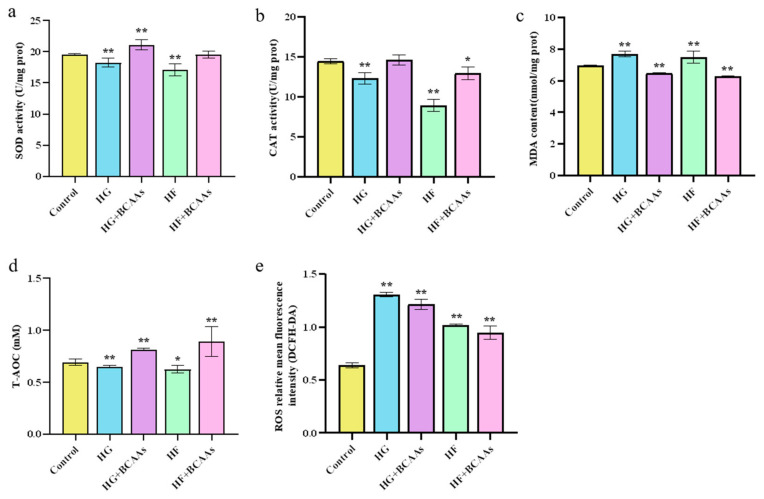
Effects of BCAAs supplementation on antioxidant capacity in hepatocytes of spotted seabass (*Lateolabrax maculatus*): (**a**) SOD activity; (**b**) CAT activity; (**c**) MDA content; (**d**) T-AOC; (**e**) ROS concentration. * indicates significant differences between groups (*p* < 0.05); ** indicates extremely significant differences between groups (*p* < 0.01).

As shown in Figure 2, BCAAs supplementation under HG or HF conditions significantly enhanced SOD activity (*p* < 0.05), and T-AOC (*p* < 0.05) in hepatocytes. Additionally, it markedly reduced MDA levels (*p* < 0.05) and decreased ROS production.

### 3.3. Cellular Metabolism

**Figure 3:**
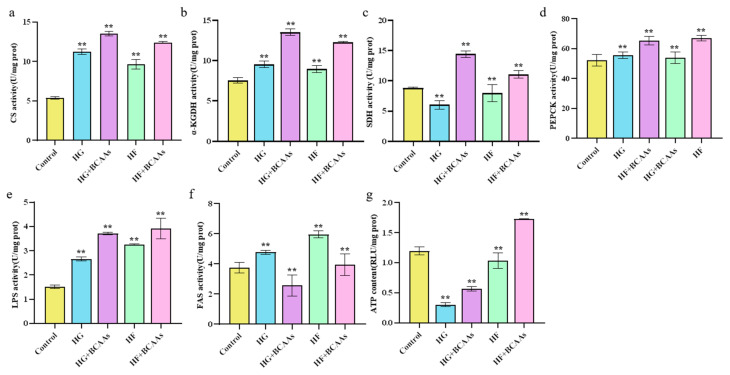
Effects of BCAAs supplementation on hepatocyte metabolism in spotted seabass (*Lateolabrax maculatus*): (**a**) CS activity; (**b**) CAT activity; (**c**) SDH content; (**d**) PEPCK activity; (**e**) LPS activity; (**f**) FAS activity; (**g**) ATP content. ** indicates extremely significant differences between groups (*p* < 0.01).

The activity of key metabolic enzymes involved in the citric acid cycle is presented in Figure 3. Under HG or HF conditions, BCAAs supplementation significantly increased the activity of CS, α-KGDHC, SDH, PEPCK, and LPS (*p* < 0.05) while reducing FAS activity (*p* < 0.05). These metabolic changes were accompanied by a significant increase in ATP content (*p* < 0.05).

**Figure 4:**
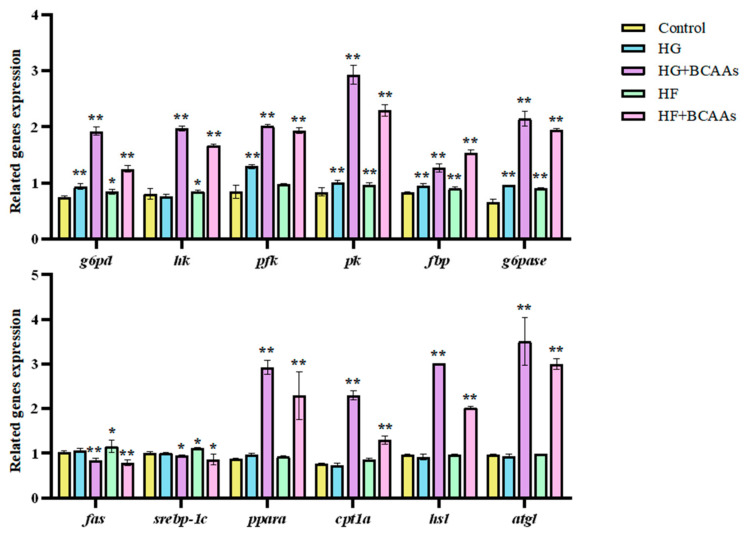
Effects of BCAAs supplementation on the expression of genes related to glucose and lipid metabolism in hepatocytes of spotted seabass (*Lateolabrax maculatus*) under high-glucose or high-fat load. * indicates significant differences between groups (*p* < 0.05); ** indicates extremely significant differences between groups (*p* < 0.01).

The expression of genes related to glucose and lipid metabolism in hepatocytes is shown in Figure 4. Compared to the control group, BCAAs supplementation significantly downregulated the expression of lipogenic genes *fas* and *srebp-1c* (*p* < 0.05), while significantly upregulating the expression of lipolytic genes *ppaα* and *atgl* (*p* < 0.05). Furthermore, genes involved in glucose metabolism, including *g6pd*, *hk*, *pfk*, *pk*, *fbp*, and *g6pase*, were significantly upregulated (*p* < 0.05).

### 3.4. Mitochondrial Function

**Figure 5:**
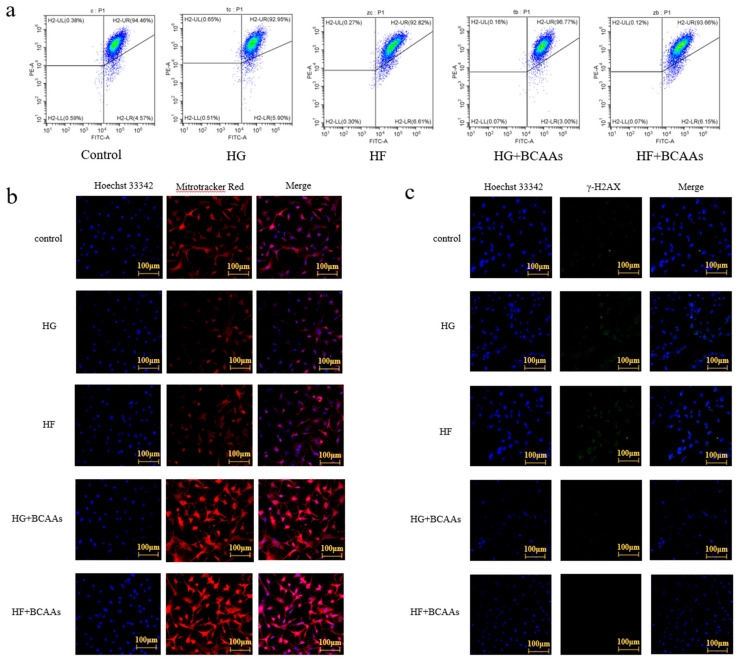
Effects of BCAAs supplementation on mitigating hepatocyte mitochondrial damage induced by high-glucose or high-fat conditions: (**a**) cell membrane potential; (**b**) mitochondrial activity; (**c**) mitochondrial DNA damage.

The effects of BCAAs supplementation on the mitochondrial membrane potential of *L. maculatus* are shown in Figure 5. BCAAs incorporation alleviated the reduction in membrane potential induced by HG or HF conditions, enhanced mitochondrial activity, and reduced DNA damage in hepatocytes.

**Figure 6:**
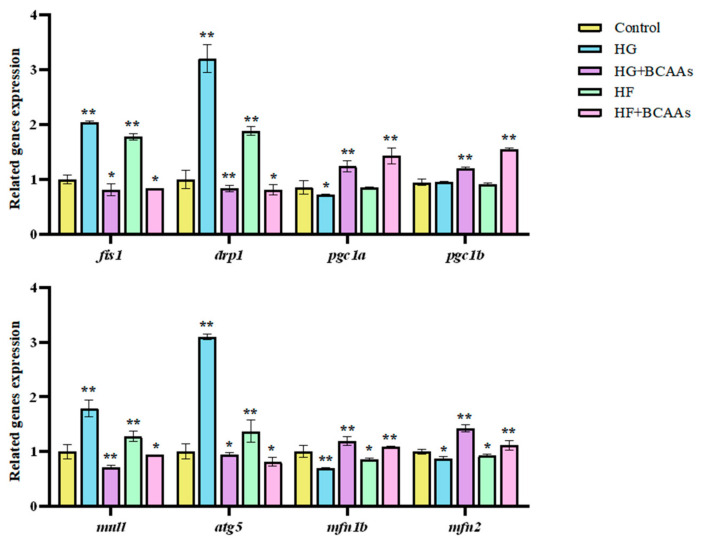
Effects of BCAAs supplementation on the expression of genes related to mitochondrial fusion and fission under high-glucose or high-fat conditions. * Indicates significant differences between groups (*p* < 0.05); ** indicates extremely significant differences between groups (*p* < 0.01).

The expression of genes associated with hepatocyte mitochondrial fusion, fission, biogenesis, and autophagy is displayed in Figure 6. BCAAs supplementation significantly promoted the expression of mitochondrial fusion genes *pgc1α* and *pgc1b*, as well as biogenesis genes *mfn1b* and *mfn2* (*p* < 0.05). Conversely, it suppressed the expression of fission-related genes *fis1* and *drp1*, along with autophagy-related genes *mul1* and *atg5* (*p* < 0.05).

## 4. Discussion

### 4.1. BCAAs Supplementation Mitigated Metabolic Disorders in Hepatocytes Induced by HG or HF Load

The liver plays a central role in metabolism in fish and other animals, making it a primary focus of metabolic research. Previous studies have shown that HG or HF diets lead to excessive glycogen and lipid deposition, resulting in liver damage [27,28]. In the present study, hepatocytes exposed to HG or HF conditions exhibited significantly elevated ALT and AST activities in the supernatant, indicating hepatocyte damage. However, BCAAs supplementation effectively alleviated lipid accumulation and glycogen deposition while reducing ALT and AST activities. These findings align with previous research in mice fed high-fructose or high-fat diets, suggesting that isolated hepatocytes exhibit metabolic responses comparable to in-vivo conditions.

Liver enzymes serve as key catalysts in physiological metabolism in fish [29]. To better understand the metabolic effects of BCAAs we analyzed the activities of key enzymes involved in the citric acid cycle, gluconeogenesis, and lipid metabolism. BCAAs supplementation significantly increased the activities of CS, α-KGDHC, SDH, PEPCK, and LPS while reducing FAS activity. The increased activities of CS, α-KGDHC, and SDH indicate enhanced glucose and lipid utilization in the citric acid cycle, while upregulated PEPCK activity, a rate-limiting enzyme in gluconeogenesis [30,31], indicates an acceleration of gluconeogenesis and reduced glycogen accumulation.

HK, G6pd, PFK, and PK are pivotal glycolytic enzymes essential for energy production. Meanwhile, FBP and G6Pase, are key regulators of gluconeogenesis, governing glycogen breakdown and glucose homeostasis [32,33,34]. Our study demonstrated that BCAAs supplemented significantly upregulated the expression of glycolysis-related enzymes (*g6pd*, *hk*, *pfk*, *pk*) and gluconeogenesis-related enzymes *(fbp*, *g6pase*). These findings suggest that BCAAs enhance both glycolysis and gluconeogenesis in hepatocytes, strengthening energy metabolism while preventing excessive glycogen accumulation.

The sterol regulatory element-binding protein (SREBP) family is a key regulator of cholesterol, fatty acid, triglyceride, and glycerophospholipid synthesis, with SREBP-1c specifically controlling the transcription of lipogenic genes such as *fas* and *acc*. FAS and acetyl-CoA carboxylase (ACC) are major enzymes involved in fatty acid biosynthesis [35,36,37]. Conversely, ATGL is crucial for lipolysis, as it catalyzes the initial break down of triglycerides. Impaired ATGL expression can lead to excessive triglyceride accumulation, contributing to obesity and metabolic disorders [38,39,40,41]. Our study revealed that BCAAs supplementation significantly downregulated the expression of lipogenic genes *fas* and *srebp-1c*, while upregulating lipolytic genes *ppaα* and *atgl*. This suggests that BCAAs promote lipid breakdown and inhibit lipid synthesis, thereby reducing fat deposition in the liver of *L. maculatus*. BCAAs likely modulate the expression of SREBP precursors, thereby reducing *srebp-1c* expression and its downstream targets *fas* and *acc*, while simultaneously enhancing the expression of lipolysis-related genes such as *atgl*, thereby improving lipid homeostasis by reducing triglyceride accumulation.

Antioxidant markers provide valuable insights into organismal health. AOC reflects the cumulative antioxidant potential from substances and enzymes, with SOD and CAT serving as key enzymes for neutralizing free radicals [42,43]. MDA, a product of lipid peroxidation, serves as a marker of oxidative stress [44,45]. In this study, HG and HF conditions caused a significant decline in T-AOC, and activities of SOD, and CAT, along with increased MDA levels, indicating oxidative stress in *L. maculatus* hepatocytes. However, BCAAs supplementation effectively alleviated oxidative stress, restoring antioxidant enzyme activity and enhancing overall cellular antioxidant capacity.

### 4.2. BCAAs Supplementation Alleviated Mitochondrial Damage Caused by HG or HF Loads

Mitochondria play a fundamental role in liver metabolism, and their structural integrity is essential for cellular function [46]. The mitochondrial membrane acts as a critical barrier, protecting the organelle from damage. However, its permeability increases significantly when mitochondria are compromised [47,48]. In this study, HG or HF exposure caused a decrease in mitochondrial membrane potential and a decline in mitochondrial activity in hepatocytes. Conversely, BCAAs supplementation preserved mitochondrial membrane potential and activity, suggesting their protective role in maintaining mitochondrial stability.

The mitochondrial respiratory chain is a primary site of ROS production. Disruption of this chain can lead to electron leakage, which combines with oxygen and other molecules to generate ROS. This study found that HG or HF conditions inhibited ATP production and increased ROS levels. However, BCAAs supplementation significantly alleviated these adverse effects.

Mitochondrial DNA (mt DNA), a circular DNA located within mitochondria, encodes proteins essential for energy metabolism. Unlike nuclear DNA, mt DNA is highly susceptible to oxidative damage caused by ROS, which can impair mitochondrial function and overall cellular health. This study demonstrated that BCAAs supplementation significantly mitigated mt DNA damage induced by high-glucose or high-fat conditions, reducing oxidative stress and preserving mitochondrial integrity in hepatocytes.

Mitochondria are highly dynamic organelles that undergo constant renewal under normal physiological conditions. Two key processes—mitochondrial biogenesis (the generation of new mitochondria) and mitochondrial autophagy (the clearance of damaged or aged mitochondria)—work together to maintain mitochondrial homeostasis [49,50]. PGC-1α and PGC-1β, are crucial transcriptional regulators of mitochondrial biogenesis. They coordinate the activation of downstream transcription factors, enhancing mt DNA transcription and the synthesis of key mitochondrial enzymes, thereby promoting mitochondrial generation [51]. Notably, PGC-1β has been found to be more effective than PGC-1α in driving mitochondrial biogenesis in fish. This study demonstrated that HG or HF exposure significantly downregulated the expression of *pgc-1α* and *pgc-1β*, while BCAAs supplementation restored their expression levels. These findings indicate that BCAAs can reverse the inhibition of mitochondrial biogenesis caused by HG or HF conditions in the liver of *L. maculatus*, thereby promoting mitochondrial generation.

Mitochondrial autophagy, regulated by the PINK1/parkin pathway, is another critical process for maintaining mitochondrial health, with genes such as *mul1* and *atg5* playing essential roles in this pathway [52]. This study revealed that HG or HF exposure significantly suppressed the expression of *mul1* and *atg5*. However, BCAAs supplementation upregulated these genes, thereby activating mitochondrial autophagy in the liver of *L. maculatus*. A balanced relationship between mitochondrial autophagy and biogenesis is vital for maintaining mitochondrial function. Disruption of this balance can impair mitochondrial homeostasis and biogenesis. The findings of this study underscore the protective effects of BCAAs in maintaining this balance, reducing mitochondrial damage, and promoting overall mitochondrial health in hepatocytes under HG or HF stress.

## 5. Conclusions

In summary, the isolated hepatocytes of *L. maculatus* exhibited physiological responses comparable to in vivo conditions under HG or HF conditions, establishing them as a reliable model for studying fish metabolism. BCAAs supplementation effectively alleviated mitochondrial damage, promoted mitochondrial biogenesis, and preserved oxidative phosphorylation processes in hepatocytes subjected to HG or HF conditions. These effects mitigated oxidative stress-induced damage to both hepatocytes and mitochondria, thereby supporting cellular metabolic homeostasis. In the future, aquaculture production can benefit from optimizing feed formulations by incorporating appropriate amounts of BCAAs. This strategy has the potential to improve feed utilization, reduce the incidence of metabolic disorders, and promote healthy growth and production efficiency in fish.

## Figures and Tables

**Table 1:** Sequences of the primers used for real-time PCR.

Target Gene	Forward Sequence (5′−3′)	Reversed Sequence (5′−3′)	Annealing Temperature
*g6pd*	ATGCTCTGTTTGGTCGCCAT	ACATCCGACAGAGCAACAGG	60
*hk*	CTGGCTTGTGGGGACAGATT	GAGGCTGGCCCTCTTTATCC	60
*pfk-1*	CGAGGGGCTAAATGTCAGGG	AAGGGGCATTCCGGTGATTT	60
*pk*	GTGGCCCAATCCAAATGTCC	GCAAGAGTGAGAGTTGGGGT	60
*fbp*	AACTGAGAAAGTCCCCCGAC	CCGGCCAAAACCTCGTATCT	60
*g6pase*	CAGGTCATGGGGTACTGCTC	TTCCCGCTTTGGTTTCACCT	60
*fas*	AAACTGAAGCCCTGTGTGCC	CACCCTGCCTATTACATTGCTC	60
*srebp-1c*	CCTCACTCTGCAGCCAATCA	CGTAGTCCCACCCTCAAACC	60
*ppaα*	CCGTGCGTGTTTTCACCATT	AGACCAAATACATCGCCCCC	60
*cpt-1α*	CCTCAATGATACATCGGAACCC	CTGCGGCTCATCATCTAACG	60
*hsl*	CGAAACACAGAGACGGTCCA	TCATGACATCTACCAGCCGC	60
*atgl*	CTTCCTCTCCGCAACAAGTC	TGGTGCTGTCTGGAGTGTTC	60
*drp1*	CTCGCCAACAGAAACGGAAC	TGGCACTTTGGTCTTCGACA	60
*mfn1b*	GTCAACGCTATGCTGAGGGA	TCATCAGAGCCCTCCGTCTT	60
*mfn2*	TTCCAACGACCCAACACCAA	GTAGGCCCCCAACTGTTCAA	60
*mul1*	GCTGCCGTGATACGAGTCAT	ACGTTGGACAAGGACTGGAC	60
*atg5*	TCAGTCGCTGCCATTAGAGC	TCTCGTCACCTGCGAAAACT	60
*pgc-1α*	AACCCGACTCTTATCCCTCC	CGTATCAACGCCACAGCAC	60
*pgc-1β*	GTTCCTCCGAACTCCCAGTG	GCAACACCCCTCCAACTACA	60
*fis1*	GTCCCGGGAGTCATCCTTTG	ACAATGAGCTGGTGAAGGGAG	60
*β*-actin	CAACTGGGATGACATGGAGAAG	TTGGCTTTGGGGTTCAGG	60

**Note:** *g6pd*, glucose-6-phosphate dehydrogenase; *hk*, hexokinase; *pfk-1*, phosphofructokinase-1; *pk*, pyruvate kinase; *fbp*, fructose 1, 6-bisphosptase; *g6pase*, glucose-6-phosphatase G-6-pase; *fas*, fatty acid synthase; *srebp-1c*, sterol regulatory element-binding protein 1c; *ppaα*, peroxisome proliferators-activated receptors; *cpt-1α*, carnitine palmitoyl transferase 1A; *hsl*, hormone-sensitive triglyceride lipase; *atgl*, adipose triglyceride; *drp1*, dynamin-related protein 1; *mfn1b*, mitofusin1b; *mfn2*, mitofusin2; *mull*, mitochondrial E3 ubiquitin protein ligase 1; *atg5*, autophagy related 5; *pgc-1α*, proliferator-activated receptor gamma co-activator 1α; *pgc-1β*, proliferator-activated receptor gamma co-activator 1β; *fis1*, mitochondrial fission protein 1; *β-actin*, beta-actin.

## Data Availability

The datasets generated and/or analyzed during the current study are available from the corresponding author upon reasonable request.
